# Real-Time Ferrogram Segmentation of Wear Debris Using Multi-Level Feature Reused Unet

**DOI:** 10.3390/s24082444

**Published:** 2024-04-11

**Authors:** Jie You, Shibo Fan, Qinghai Yu, Lianfu Wang, Zhou Zhang, Zheying Zong

**Affiliations:** 1 Ocean College, China University of Geosciences Beijing, Beijing 100083, China; hhan0703@163.com; 2 School of Information Engineering, China University of Geosciences Beijing, Beijing 100083, China; 15547227511@163.com; 3 Alumni and Social Cooperation Office, China University of Geosciences Beijing, Beijing 100083, China; 4 College of Mechanical & Electrical Engineering, Inner Mongolia Agricultural University, Hohhot 010018, China; wanglianfu1126@foxmail.com (L.W.); zhangzhou0416@163.com (Z.Z.)

**Keywords:** debris image, semantic segmentation, convolutional neural network

## Abstract

The real-time monitoring and fault diagnosis of modern machinery and equipment impose higher demands on equipment maintenance, with the extraction of morphological characteristics of wear debris in lubricating oil emerging as a critical approach for real-time monitoring of wear, holding significant importance in the field. The online visual ferrograph (OLVF) technique serves as a representative method in this study. Various semantic segmentation approaches, such as DeepLabV3+, PSPNet, Segformer, Unet, and other models, are employed to process the oil wear particle image for conducting comparative experiments. In order to accurately segment the minute wear debris in oil abrasive images and mitigate the influence of reflection and bubbles, we propose a multi-level feature reused Unet (MFR Unet) that enhances the residual link strategy of Unet for improved identification of tiny wear debris in ferrograms, leading to superior segmentation results.

## 1. Introduction

The stable operation and prevention of sudden failures in machinery and equipment are crucial in the field of modern industry. Tribo-pair wear failure stands out as a primary cause for mechanical equipment failures, accounting for over half of such occurrences. In particular, the wear failure of these key components, namely bearings and gears [1], constitutes a significant proportion of mechanical equipment failures. Excessive wear not only causes damage to the key components within the equipment, but also escalates operational costs and poses potential risks of injury and property damage. To mitigate these issues, vibration monitoring and oil monitoring have emerged as indispensable measures [2,3]. Despite its ability to rapidly acquire signals and identify issues with friction pairs to a certain extent, vibration monitoring is susceptible to external excitation interference and resonance distortion, and lacks the necessary sensitivity for the early detection of mechanical equipment wear failures. Therefore, there is an imperative for the development of a more robust and highly sensitive monitoring methodology capable of pre-emptively detecting potential equipment malfunctions.

Wear debris generated by mechanical tribo-pairs contains crucial information regarding the operation of mechanical equipment [4,5]. The shape, size, color, texture, and other characteristics of wear fragments directly manifest the wear state of the tribo-pair [6,7,8]. According to statistical data, the size of wear particles is typically maintained within the range of 10–20 μm during normal machine operation [9,10]. The size of wear particles will increase to 50–100 μm and the concentration of wear particles will significantly rise when mechanical equipment parts undergo progressive abnormal wear [11,12]. Wear debris analysis plays an increasingly pivotal role in the prognosis and diagnosis of mechanical systems, thus giving rise to numerous associated methodologies, such as atomic emission spectroscopy, particle counting, and ferrography [13,14].

The technology known as ferrography is employed to assess the wear condition of mechanical equipment by analyzing the wear particles present in lubricating oil. During the operation of mechanical equipment, friction between components generates wear particles that contaminate the lubricating oil. These wear particles carry valuable information pertaining to the condition of the mechanical system. By conducting an analysis of the wear particles present in lubricating oil, ferrography has the capability to extract and identify the morphological characteristics exhibited by these particles. Distinct wear modes give rise to wear particles with varying shapes [15], and these characteristics are employed for assessing the degree of equipment tribo-pair wear [16]. Furthermore, the application of ferrography analysis technology enables the identification and localization of abnormal wear through the comprehensive analysis and differentiation of wear particle composition [17]. Therefore, ferrography analysis technology is extensively utilized not only for monitoring wear conditions and diagnosing faults, but also for investigating wear mechanisms, assessing lubricant conditions, predicting wear trends, prognosticating failures, estimating life expectancies, and analyzing the reliability of mechanical components.

The online visual ferrograph (OLVF) is an exemplary technology derived from the conventional ferrograph [18,19]. In practical applications, OLVF is positioned externally to the lubrication system and effectively captures minuscule wear particles in the lube oil through the utilization of a high-gradient-magnetic-field force. Subsequently, micrographs are captured to acquire images of the wear particle deposition spectrum, and subsequently visual characteristics are extracted [20]. The image processing technology is utilized for particle differentiation and size identification [21], facilitating the diagnosis and assessment of machine operating wear conditions [22], as well as enabling effective monitoring of machine wear conditions. The index of particle coverage area (IPCA) is employed in this process to characterize particle concentration and analyze the characteristics of large debris within the spectrum, enabling the identification of abnormal wear. OLVF has been successfully implemented in various practical applications, encompassing high-power gearboxes, diesel engines, gasoline engines, marine gas turbines, and even aero engines.

The OLVF ferrogram is available in two forms, namely the reflection ferrogram (RF) and the transmission ferrogram (TF). The RF is acquired by illuminating the sample with reflected light, and its imaging principle involves positioning the light source above the debris deposition field. The TF is acquired by illuminating the sample with a transmitted light source. Its imaging principle involves positioning the light source within the abrasive deposition field. In the monitoring of wear in OLVF, the uncertainty surrounding the shape of wear particles results in their accumulation and adhesion when they are large, while their dispersion in oil makes it difficult to capture them when they are small. The two imaging methods result in intricate light distribution on the surface, with a conspicuous contrast between light and dark. Moreover, due to the interference of bubbles, wear particle segmentation becomes exceedingly challenging. Consequently, numerous algorithms have been developed in response to this issue.

In recent years, deep learning algorithms have been extensively employed for the segmentation of grain chains and extraction of grain signs in ferrographic images. For example, Wang et al. [23] proposed a hybrid approach combining marker-controlled watershed segmentation with an enhanced gray clustering algorithm to effectively segment both coarse and fine wear fragments that are deposited on the chain. In addition, Wang et al. [24] combined the watershed algorithm with the improved ant colony clustering algorithm to achieve accurate segmentation of abnormal wear fragments. Wu et al. [25] proposed an algorithm based on multi-scale morphological operation based on gray-scale and comprehensive morphological characteristics of wear fragments to achieve the segmentation of wear fragment chains. Han Lang et al. [26] proposed a binary method for wear particle images based on uniform discrete curve transformation. By using UDCT to conduct multi-scale analysis of ferrograms and nonlinear transformation of UDCT coefficients, the wear particle image was suppressed at low frequency and denoised at high frequency to solve the problem of air bubbles in lube oil interfering with wear particle recognition. Peng et al. [27] proposed an overlapping debris segmentation network to address the issue of abrasive chain overlap in ferrographic images.

The majority of existing segmentation algorithms are primarily designed for offline ferrogram analysis, characterized by high resolution, uniform illumination, significant foreground–background contrast, and an absence of bubble interference. In contrast, the presence of light and bubble interference in OLVF is inevitable, posing challenges to segmentation and rendering existing methods ineffective in accurately segmenting wear debris in OLVF.

In order to solve the above problems, this paper adopts a variety of semantic segmentation models, including DeepLabV3+, PSPNet, Segformer, and Unet, and conducts comparative experiments. We found that as the depth of the network increases, the content of feature extraction by the model becomes increasingly abstract and less specific. At this point, the model’s extraction of information becomes uncontrollable. We introduce residual networks for reusing feature information, correcting the deviation of information flow in the model, preventing the model from paying excessive attention to unnecessary information, and thus optimizing the overall segmentation information flow to enhance its segmentation capability. Finally, the MFR Unet network is proposed to achieve RF segmentation. Currently, according to the actual data and segmentation results, no potential limitations or challenges have been identified.

## 2. Proposed Method

### 2.1. MFR Unet

The Unet proposed by Ronneberger et al. [16] has demonstrated remarkable efficacy in addressing the segmentation challenges of small-sample medical images with intricate backgrounds. Unet comprises a compression channel at the front end and an extended channel at the back end. The compression channel at the front end is utilized for subsampling, comprising a convolutional layer and a pooling layer. The convolutional layer is utilized for feature extraction, followed by upsampling through the extended channel comprising an upsampling layer to restore the feature map size and a convolutional layer to recover image feature information. Upon completion of upsampling, the desired segmentation image is generated.

**Figure 1:**
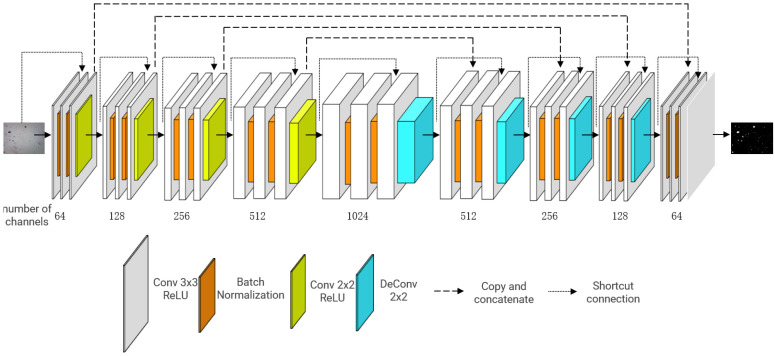
The architecture of the MFR Unet.

Accordingly, we propose the multi-level feature reused Unet (MFR Unet), as shown in Figure 1.

The residual connection is first introduced, and the connection strategy is modified. In the encoder part, an additive residual connection is employed to mitigate the issue of gradient vanishing. The input of each level is combined with the output of its corresponding convolutional layer to establish a residual connection, thereby facilitating network training. At the same time, the residual connection can make the input information easier to propagate in the network, thus improving the model performance. The decoder part establishes a concatenate connection between the corresponding levels of the encoder and decoder, effectively preserving feature information from different levels and enhancing the model’s representational capacity.

Batch normalization is employed in this study. Incorporating a batch normalization layer after each convolutional layer not only enhances the efficiency and stability of model training, but also serves as a preventive measure against overfitting. The eigenvalue distribution at each layer gradually converges towards the upper and lower bounds of the activation function’s output interval as the network depth increases or during the training process. Consequently, this phenomenon gives rise to gradient vanishing, impeding network convergence. By incorporating a batch normalization layer prior to the activation function for input normalization, the distribution of input values in each neural network layer is aligned with a standard normal distribution (mean 0 and variance 1). This alignment compels any deviating distributions to converge towards the standard distribution, thereby ensuring that activation inputs fall within the sensitive region of the nonlinear function, increasing the gradient and avoiding the problem of gradient disappearance. At the same time, it accelerates the speed of learning convergence.

In the enhanced model, a convolutional layer with a stride of 2 is employed to replace the conventional Unet maximum pooling layer (MaxPooling2D) for achieving downsampling. This approach effectively preserves a greater amount of information, thereby enhancing the accuracy and precision of image segmentation. Moreover, employing Conv2DTranspose for upsampling in the extended channel demonstrates superior capability in recovering local image features compared to conventional Unet. By using ReLU activation functions and normal distribution initialization, the model is easier to train and converges faster. The Sigmoid activation function is employed in the output layer, which is well suited for binary classification tasks and effectively constrains the output within the range of 0 to 1, thereby satisfying the requirements stipulated in this study.

Unet and its variants have certain advantages in interpretability because of their relatively simple structure, making it easy to understand the function and role of each module. By forcing the model to deeply understand essential features through residual connections, in the MFR Unet model, the multi-scale feedback mechanism provides a way to understand how the model makes segmentation decisions. Through multi-scale feedback, the model can process features multiple times at different resolutions, extracting richer information and segmenting targets at different scales. This mechanism enables the model to focus on features at different scales, thereby better capturing the details and global information of wear debris.

The MFR Unet architecture integrates technologies such as residual connections, batch normalization, and enhanced downsampling strategies. It enhances the capability to retain fine details in images, specifically targeting the characteristic fine wear debris in RF images. As a result, it demonstrates superior performance in handling complex image segmentation tasks and can be widely applied in scenarios requiring binary segmentation of wear debris. Leveraging the inherent simplicity and strong generalization capability of Unet, it also enables potential transplantation into various domains.

### 2.2. Prepare Training Data

#### 2.2.1. OLVF Ferrograms

**Figure 2:**
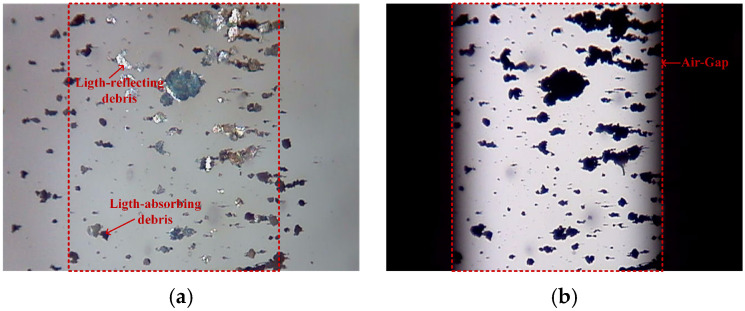
Ferrogram labeling: (**a**) reflection ferrogram (RF); (**b**) transmission ferrogram (TF).

OLVF simultaneously monitors wear and captures RFs (Figure 2a) and TFs (Figure 2b). TFs exhibit a high contrast between the wear debris and the background, with clear edges that facilitate easy segmentation. However, it is unable to display wear debris deposited outside the air gap area in TFs. Compared to TFs, RFs exhibit a wider visible range and provide richer visual information; however, the edge contrast of wear debris is lower, posing challenges for segmentation. To generate training data, we collected 1725 RFs and 1725 TFs with dimensions of 640 pixels × 480 pixels.

The data we used were collected from the complete operating cycle of the gear test bench, and the images of wear debris in the dataset were sampled from these data. The data are highly representative and can encompass the specific conditions of the gear at each cycle of its life span.

In the operation of machinery, from initially healthy gears to ultimately damaged ones, the shapes, sizes, and other characteristics of wear debris vary, demonstrating diversity and heterogeneity in debris. This encompasses various scenarios of wear debris. The results are not influenced by specific experimental settings or the dataset.

#### 2.2.2. Image Characterization of RFs

As shown in Figure 2a, the debris is encapsulated within the lubricating oil during the wear debris imaging conducted by OLVF, and the RFs are subject to many interferences caused by light, oil transparency, etc. In the RFs, the presence of either darker or brighter wear debris affects the contrast difference between the debris and background, potentially leading to missed detection during debris segmentation. Accordingly, the presence of light-reflecting debris within RFs constitutes a primary factor contributing to the inaccuracies observed in binarization.

**Figure 3:**
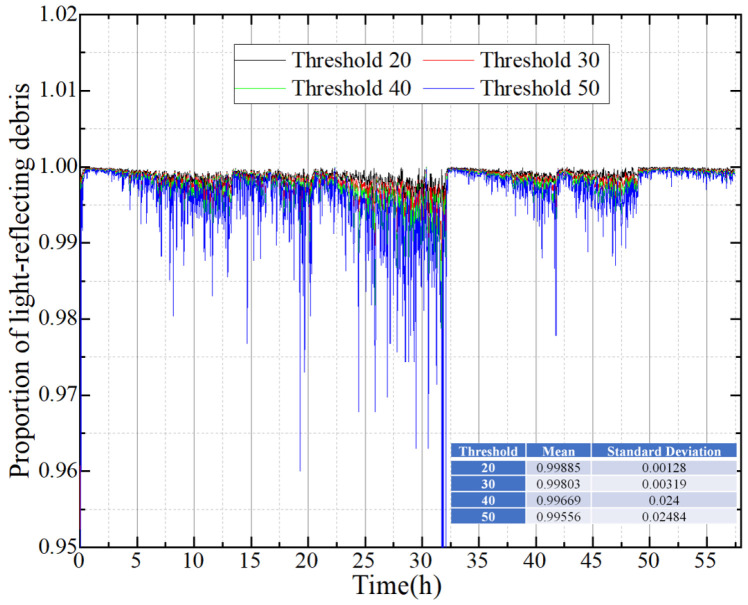
Statistical results of light-reflecting debris in the air gap of RFs.

The ratio of the coverage area of light-reflecting debris in the air gap region to the total reflective coverage area was computed from the 1725 RFs. The RFs were binarized using different thresholds, as illustrated in Figure 3. The statistical findings demonstrate that the proportion of light-reflecting debris in the air gap consistently exceeded 99.5%. The light-reflecting debris of RFs is primarily concentrated in the air gap region, with negligible amounts outside. Fortunately, binarized TFs can compensate for this portion of RFs. Therefore, TFs are introduced to improve the binarization accuracy of RFs so as to realize the automatic marking of RFs.

#### 2.2.3. Automatic Labeling of RFs Combined with TFs

The morphological characteristics of wear debris are derived from the edges of the debris, necessitating pixel-level marking of the wear debris. However, the intricate nature of wear debris edge characteristics poses significant challenges for manual labeling. To address this issue, an automated approach is proposed, comprising two distinct steps:

(1) Excluding ferrograms affected by bubble interference, retain RFs and TFs with high imaging quality, and enhance the accuracy of labeling.
(2) The automatic threshold method is employed for the segmentation of RFs and TFs, followed by the superimposition of the two segmentation outcomes to generate binary masks.

The mapping relationship between RFs and binary masks is established to achieve automatic labeling of the RFs. Importantly, the RF serves as the input to the network while the TF assists in annotation and enhances annotation accuracy.

### 2.3. Model Evaluation Criteria

This paper uses three indexes, including accuracy, mean pixel accuracy (MPA), and mean intersection over union (mIoU), to evaluate the model.

Accuracy measures the model’s ability to correctly classify all samples, which can be calculated as follows:

(1) $$Accuracy=\frac{TP+TN}{TP+TN+FP+FN}$$

where TP (true positive) refers to the count of accurately predicted positive samples among those that are truly positive; TN (true negative) is the number of samples that are actually negative and are correctly predicted to be negative; FP (false positive) is the number of samples that are actually negative but are incorrectly predicted to be positive (false positive); and FN (false negative) represents a false negative, that is, the number of samples that are actually positive but are incorrectly predicted to be negative (missed). 

MPA measures the ability of the model to correctly classify each pixel, that is, the ratio of the number of correctly classified pixels to the total number of pixels. MPA can be calculated as follows:

(2) $$MPA=\frac{1}{N}\sum_{i=1}^{N}\frac{{TP}_{i}}{{TP}_{i}+{FP}_{i}}$$

where N is the total number of images; ${TP}_{i}$ is the number of correctly classified pixels in the i-th image (true positives); and ${FP}_{i}$ is the number of false positives in the i-th image.

The mIoU metric incorporates the model’s classification accuracy across various categories and quantifies the degree of overlap between the predicted segmentation area and the ground-truth label. mIoU is particularly suitable for tasks involving pixel-level classification. Its calculation can be expressed as follows:

(3) $$mIoU=\frac{1}{c}\sum_{i=1}^{c}\frac{{TP}_{i}}{{TP}_{i}+FP+{FN}_{i}}$$

where ${FN}_{i}$ is the number of pixels in the first category that are misclassified as other categories (false negatives).

This study aims to investigate the performance and efficiency of various deep learning models for the binary segmentation task of wear debris in lube oil through comprehensive analysis and comparison. Five models, namely, DeepLabV3+, PSPNet, Segformer, Unet, and MFR Unet, were trained and their performances were compared.

The aforementioned five models were trained and their performance on the test set was compared. Using Anaconda, Python 3.8, the specific environment consists of the following: scipy 1.2.1; numpy 1.17.0; matplotlib 3.1.2; opencv 4.1.2.30; torch 1.2.0; torchvision 0.4.0; tqdm 4.60.0; Pillow 8.2.0; h5py 2.10.0.

**Figure 4:**
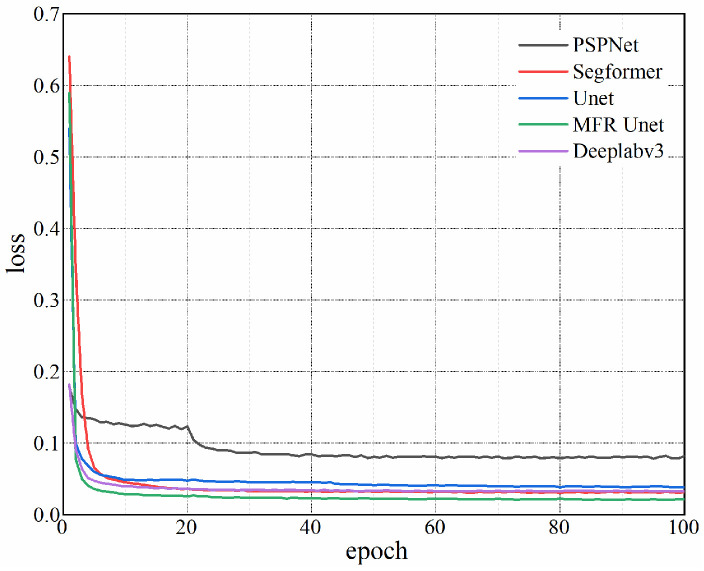
Loss curves of five network models.

As shown in Figure 4, the loss curves corresponding to 100 epochs were obtained, revealing that MFR Unet exhibits a superior convergence rate. The mIoU, MPA, and accuracy of each model on the test set were computed in addition to being depicted in Figure 4 and Table 1. Notably, MFR Unet outperformed other models in terms of mIoU, MPA, and accuracy. The MFR Unet has demonstrated remarkable advantages in terms of both performance and efficiency, thereby holding great potential to offer more dependable support for practical applications such as equipment monitoring and maintenance.

**Figure 5:**
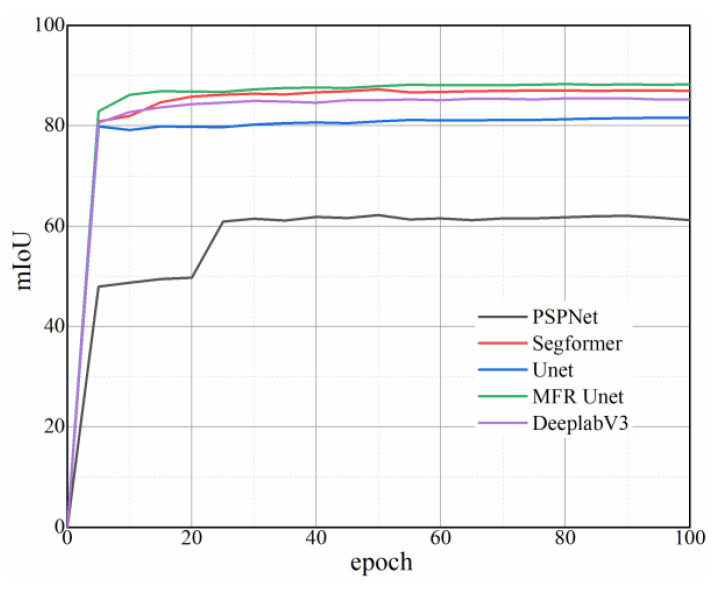
Epoch–mIoU curves of five network models.

As shown in Figure 5, optimization is conducted through the loss function, learning rate, and other factors, with a greater emphasis on segmentation accuracy in this process. However, there is no blind increase in model complexity to improve the effectiveness.

## 3. Segmentation Results and Discussions

### 3.1. Small Debris Recognition of MFR Unet

**Figure 6:**
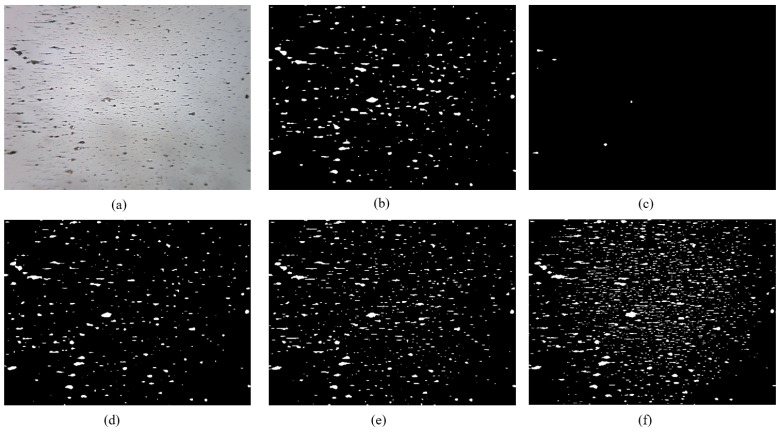
Segmentation of small debris: (**a**) original; (**b**) DeepLabV3+; (**c**) PSPNet; (**d**) Segformer; (**e**) Unet; (**f**) MFR Unet.

Oil debris generated by certain mechanical equipment, such as pumps, valves, and aircraft engines, exhibits a small particle size. Furthermore, the quantity of small debris serves as a direct indicator of the wear rate of the tribo-pairs, and the precise and effective identification of these minute wear particles forms the foundation of quantitative wear analysis. The segmentation of RFs containing a lot of small wear debris was performed using five models, as illustrated in Figure 6.

The DeepLabV3+ features a deep variable convolutional network structure, capable of capturing a wider range of contextual information. It utilizes dilated convolutions to expand the receptive field, which helps in handling large-scale targets. However, the training and inference processes are relatively complex, requiring more computational resources. It may not be sensitive enough to small targets or fine details.

The PSPNet utilizes pyramid pooling modules to capture contextual information at different scales, aiding in handling wear debris with large variations in scale. It has good memory and computational efficiency. However, it may not handle boundary details finely enough and may not perform well on wear debris with complex textures or structures.

The Segformer captures global contextual information through a global self-attention mechanism, aiding in handling large-scale wear debris. It has high parallelism and can effectively utilize multiple GPUs for training. However, it has high computational complexity, leading to longer training times, and may not be sensitive enough to small targets.

The Unet performs well on small datasets and has strong generalization ability. It is suitable for segmentation tasks on small-scale targets. However, its segmentation performance may be poor for large-scale targets, and it lacks a mechanism to capture global contextual information.

The MFR Unet introduces a multi-scale feedback mechanism, aiding in extracting features of different scales, suitable for handling wear debris with large variations in scale. It combines the simplicity of Unet with the ability to capture multi-scale information. It may face overfitting issues, especially with limited training data.

PSPNet demonstrated limited efficacy in detecting such minute wear debris, resulting in the identification of only a minimal quantity. The segmentation performances of DeepLabV3+, Segformer, and Unet exhibit similarity in effectively segmenting large wear debris; however, these three models still demonstrate limitations in accurately identifying very small wear debris with low contrast. The MFR Unet model exhibited superior capability in detecting small wear debris compared to the other four models. By ensuring the high-quality segmentation of large wear particles, it is possible to significantly enhance the identification accuracy of small wear debris, thereby facilitating the quantitative analysis of lubricating oil wear debris. This advancement in analysis techniques contributes to early problem detection and enables proactive measures.

### 3.2. Segmentation of Ferrograms Containing Bubbles

**Figure 7:**
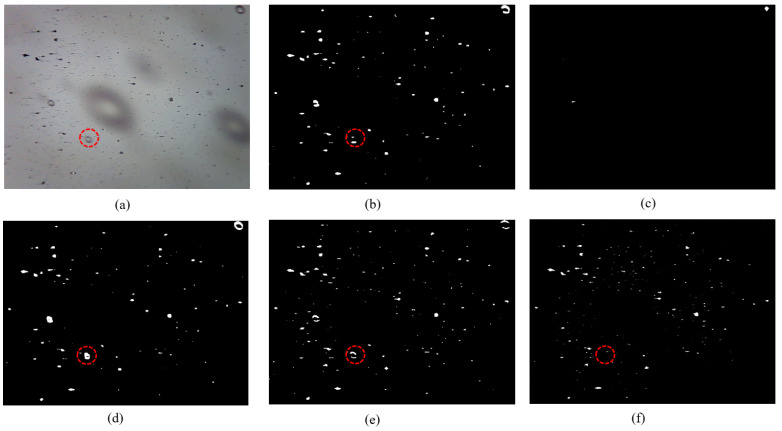
Segmentation of ferrograms containing bubbles: (**a**) original; (**b**) DeepLabV3+; (**c**) PSPNet; (**d**) Segformer; (**e**) Unet; (**f**) MFR Unet.

During the interaction between the tribo-pairs and the lubricating oil, air is entrained into the lubricating oil, resulting in bubble formation. This phenomenon is commonly observed, for instance, in gearbox systems that are lubricated by an oil bath. The bubbles flow through the OLVF and create smooth-edged blob shadows, which can be easily recognized as wear debris by traditional segmentation algorithms. This seriously affects the accuracy of wear debris recognition. This is a common challenge encountered by all image-based debris detectors, including the five network models depicted in Figure 7. While PSPNet fails to segment wear debris, it also does not address the segmentation of bubbles. DeepLabV3+, Segformer, and Unet exhibit effective identification of large bubbles, but still misclassify some small bubbles as wear debris. The MFR Unet demonstrates robustness in handling bubbles of varying scales, effectively mitigating the impact of bubbles on wear debris segmentation results and enhancing the model’s ability to accurately focus on wear debris.

### 3.3. Retention of Debris Edge in MFR Unet

**Figure 8:**
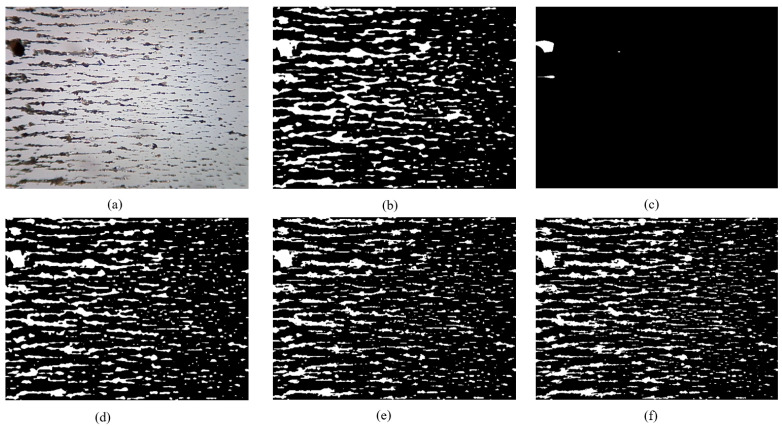
Edge preserving of wear debris: (**a**) original; (**b**) DeepLabV3+; (**c**) PSPNet; (**d**) Segformer; (**e**) Unet; (**f**) MFR Unet.

The wear debris edge serves as a primary source of information for extracting wear debris features, while the retention of this edge represents a crucial evaluation criterion for wear debris algorithms. The segmentation performance of PSPNet is limited in effectively capturing the wear debris, resulting in minimal delineation of the wear debris edges, as depicted in Figure 8. The edge retention of DeepLabV3+ exhibits suboptimal performance, with evident manifestations of overfitting at the periphery of the wear debris. The Segformer model also exhibits the phenomenon of debris edge overfitting; however, it outperforms DeepLabV3+ in terms of segmentation effectiveness. The Unet and MFR Unet models effectively preserve the edges of wear debris and accurately capture their morphological characteristics, thereby establishing a solid foundation for feature extraction.

## 4. Application in Gearbox Monitoring

**Figure 9:**
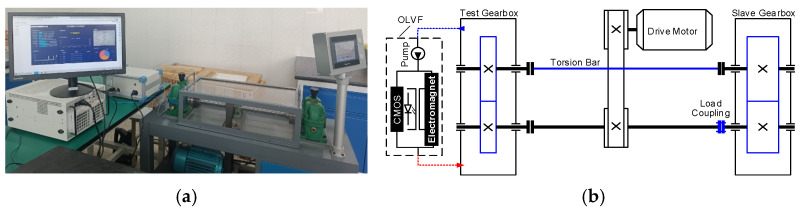
Schematic diagram of the OLVF monitoring gearbox: (**a**) Photograph. (**b**) Schematic.

A gear wear monitoring experiment was conducted to validate the efficacy of the developed methodology, wherein an accelerated gear wear experiment was performed on a dedicated test bench for gear wear. The test bench comprised two oil-bath-lubricated spur gearboxes, as illustrated in Figure 9. An AC three-phase electrical motor (3 KW) drove the test bench at a speed of 1450 rpm. The parameters of the test gear are presented in Table 2. The load applied to the wheel was 200 Nm. During the test, downtime sampling was performed to collect oil samples for offline analysis. Sampling intervals were approximately 6 h in the early stages of the test and decreased to approximately 3 h in the later stages of the test.

**Figure 10:**
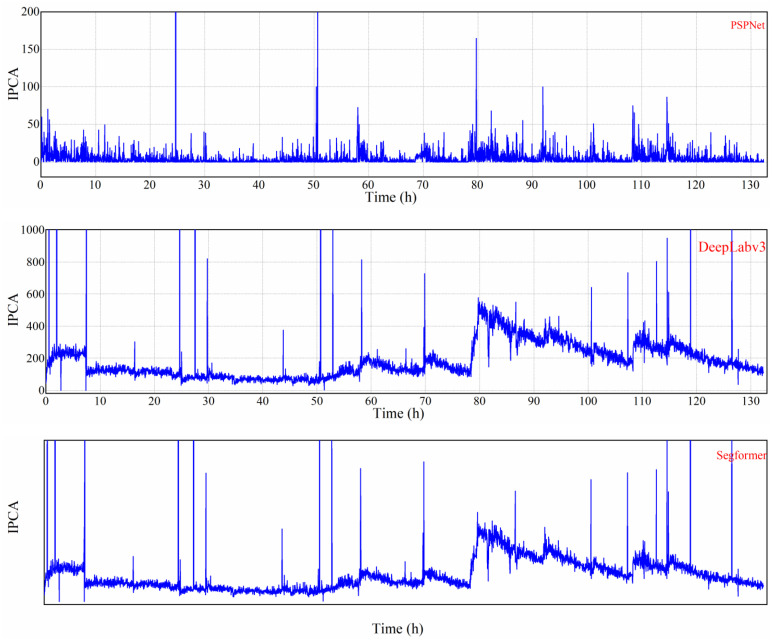

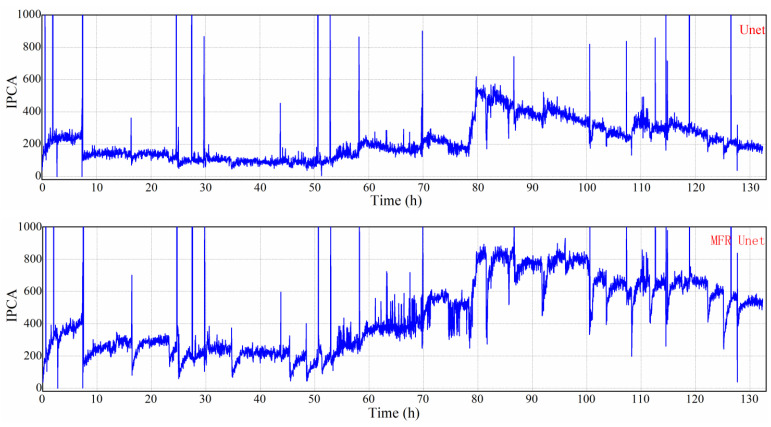
Comparison of IPCA variation curves calculated from five network models.

The monitoring of on-line wear debris concentration was conducted using OLVF, positioned in the gearbox near the oil level. The OLVF effectively captured and discharged in-use oil carrying wear debris back into the gearbox. A high-resolution industrial camera within the OLVF recorded an RF and a TF every 2 min, resulting in a total sampling interval of 132 h and the acquisition of 3961 ferrograms. Five models were employed for precise segmentation of the wear debris, while the IPCA, an indicator of debris concentration, was calculated as follows [6]:

(4) $$IPCA=\frac{\sum D_{i}}{L\times W}$$

where L and W represent the length and width of the rectangular region, and $\sum D_{i}$ represents the total coverage area of the wear debris. The IPCA curves are presented in Figure 10.

Obviously, PSPNet is unsuitable for ferrogram segmentation as it can only detect a limited amount of large debris, and the IPCA curve fails to accurately represent the actual gear wear process. DeepLabV3+, Segformer, and Unet exhibit similar segmentation effects with comparable trends in the obtained IPCA curves. However, these models have limited recognition capabilities for small wear debris resulting in a significant loss of particle information in the segmentation results. In contrast, MFR Unet demonstrates distinct trends in its IPCA curve. It exhibits a superior ability to identify small particles with correspondingly larger IPCA values. Consequently, MFR Unet outperforms other models by identifying a greater number of wear particles and producing an IPCA curve that closely aligns with the actual gear wear state.

**Figure 11:**
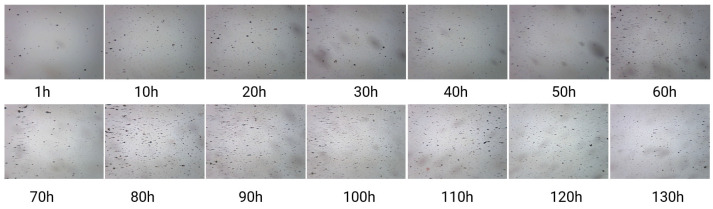
RF showing the variation in gear wear on the test bench over time.

Figure 11 is an RF showing the variation in gear wear on the test bench over time. It corresponds to the IPCA data statistics in Figure 10. After 80 h, the RF graph shows an increase in the amount of wear debris, accompanied by small wear debris. Therefore, the MRF Unet, which performs better in segmenting fine wear particles, performs better after 80 h.

## 5. Conclusions

In RFs, the wear debris exhibits varying shades compared to the background, with some appearing darker and others brighter. However, conventional segmentation methods may overlook wear debris that closely resembles the background color, leading to reduced efficiency in monitoring OLVF. Consequently, the MFR Unet model was developed specifically for RF segmentation and subsequently validated in the gear wear monitoring experiment. The inclusion of TF is dispensable for OLVF, thereby streamlining the hardware and software components of the OLVF wear monitoring system. The statistical findings demonstrate that over 99.5% of the light-reflecting particulate matter in radio-frequency fields predominantly exists within the air gap region. The present study proposes an automatic labeling approach for RFs combined with TFs, aiming to enhance the training efficiency of MFR Unet. Moreover, in comparison with the four mainstream network models, the findings demonstrate that MFR Unet exhibits a remarkable precision in wear particle segmentation, exceptional recognition capability for small-sized wear debris, an accurate identification of bubbles, and an excellent preservation of wear debris edges. These attributes are instrumental in enhancing the extraction of morphological characteristics and pattern recognition of wear debris.

## 6. Future Work

In practical applications, the quality of lubricating oil deteriorates over time, typically evidenced by changes in color, such as yellowing or darkening, which may potentially affect the final results. Regarding the dataset, we will create a dataset containing wear debris under conditions of darker lubricating oil color. Additionally, we will explore normalization techniques at different positions in the model to reduce data oscillation and improve the robustness of wear debris segmentation. We will also attempt model lightweighting by performing layer removal operations on Unet. Alternatively, we may integrate the model with transformer to further enhance its accuracy.

## Figures and Tables

**Table 1:** Accuracy and MPA of five models.

	PSPNet	DeepLabV3+	Segformer	Unet	MFR Unet
Accuracy (%)	95.91	98.41	98.61	98.14	98.76
MPA (%)	65.06	90.28	90.75	82.27	91.39

**Table 2:** Details of test gears.

Parameter	Pinion	Wheel
Number of teeth	21	82
Helix angle (deg)	11	
Normal modulus (Mn)	1.5	
Addendum modification	+0.5	+0.419
Face width (mm)	10	32
Material	20CrMnMo	
Hardness (HRC)	58	
Center distance (mm)	80	

## Data Availability

The data presented in this study are not available due to privacy.
